# Human-Centred Perspectives on Artificial Intelligence in the Care of Older Adults: A Q Methodology Study of Caregivers’ Perceptions

**DOI:** 10.3390/bs15111541

**Published:** 2025-11-12

**Authors:** Seo Jung Shin, Kyoung Yeon Moon, Ji Yeong Kim, Youn-Gil Jeong, Song Yi Lee

**Affiliations:** 1 Department of Counselling and Coaching, Dongguk University-Seoul, 30, Pildong-ro 1 gil, Jung-gu, Seoul 04620, Republic of Korea; 2 Dharma College, Dongguk University-Seoul, 30, Pildong-ro 1 gil, Jung-gu, Seoul 04620, Republic of Korea; 3 School of Interdisciplinary Studies, Dongguk University-Seoul, 30, Pildong-ro 1 gil, Jung-gu, Seoul 04620, Republic of Korea

**Keywords:** caregiving, AI virtual human, subjective perception, technology acceptance, Q methodology

## Abstract

This study used Q methodology to explore and categorise caregivers’ subjective perceptions of artificial intelligence (AI)-powered ‘virtual human’ (AVH) devices in caring for older adults. We derived 123 initial statements from literature and focus groups and narrowed them to 34 statements as the final Q sample. Seventeen caregivers, nurses, and social workers completed the Q-sorting procedure. Using principal component analysis and Varimax rotation in Ken-Q, we identified three perception types: Active Acceptors, who emphasise the devices’ practical utility in patient communication; Improvement Seekers, who conditionally accept the technology while seeking greater accuracy and effectiveness; and Emotional Support Seekers, who view the device as a tool for emotional relief and psychological support. These findings suggest that technology acceptance in caregiving extends beyond functional utility. It also involves trust, affective experience, and interpersonal interaction. This study integrates multiple frameworks, including the Technology Acceptance Model (TAM), the Unified Theory of Acceptance and Use of Technology (UTAUT), Science and Technology Studies (STS), and Human–Machine Communication (HMC) theory, to provide a multifaceted understanding of caregivers’ acceptance of AI technology. The results offer valuable implications for designing user-centred AI care devices and enhanced emotional and communicative functions.

## 1. Introduction

In April 2025, South Korea entered a super-aged society, with individuals aged 65 and older accounting for approximately 10.46 million—20.45% of the total registered population of 51.17 million (Ministry of the Interior and Safety, 2025). This demographic shift extends beyond numbers, creating structural challenges for welfare systems, the economy, and society and demanding a transition to a more sustainable social infrastructure.

As the older population continues to grow, the issue of caregiving becomes increasingly critical (OECD, 2023). In the Korean context, older adult care primarily occurs in long-term care hospitals. These facilities are specialised medical institutions that serve older adult patients requiring continuous medical management and rehabilitation following acute care. Traditionally, the responsibility of caring for aging parents rested on the eldest son, reflecting strong Confucian family values. However, as family structures have modernised and chronic illnesses among older adults have increased, this tradition has gradually shifted toward institutional care, with long-term care hospitals becoming the primary setting for supporting older adults who are physically ill (Na, 2021). In these hospitals, physicians, nurses, and caregivers collaborate within an integrated medical–care model that provides medical services and daily living support.

This study broadly defines older adult care as the provision of physical, emotional, and social support for older adults across diverse contexts, including home, community, and institutional settings, rather than restricting it to nursing homes. Within this context, ‘caregivers’ refers to professional care providers such as nurses, nursing assistants, and social workers who deliver daily care and emotional support to older adults in institutional and community-based environments. This study uses the term ‘caregiver’ in a broad and narrow sense. Broadly, it encompasses all professional care providers involved in caring for older adults. In a narrow sense, it refers to certified long-term care workers or nursing assistants who hold national qualifications in South Korea. Accordingly, this study focuses on how these professional caregivers perceive and experience artificial intelligence (AI)-powered ‘virtual human’ (AVH) systems within the context of human-centred older adult care.

Older adults often experience chronic and complex conditions, requiring long-term care that family members often provide. However, this arrangement imposes significant physical and psychological burdens on caregivers. The increasing prevalence of chronic and severe illnesses can lead to profound emotional distress for caregivers, sometimes culminating in tragic incidents such as caregiver-perpetrated violence (Choi, 2022). In response, society has begun to recognise care for older adults as a collective responsibility rather than a private obligation.

This shift, known as the ‘socialisation of elderly care’, transfers caregiving responsibilities from individuals and families to public systems (Lee, 2018). As a result, long-term care facilities and professional caregivers now play critical roles as part of the welfare infrastructure, providing medical assistance and emotional support. However, despite this evolution, caregivers continue to face excessive physical labour and emotional strain. Chronic understaffing and heavy workloads further intensify burnout and reduce the quality of care (Y. Kim & Yeo, 2018).

In response to these challenges, AI-driven care technologies have emerged as promising solutions (Lee et al., 2023). Voice-enabled systems integrated with generative pre-trained transformer (GPT)-based language models are gaining attention for their potential to improve accessibility and facilitate natural human–computer interaction (HCI) (Cao et al., 2024). These systems can engage in flexible, open-ended conversations, offering users a human-like interaction experience (Edwards & Edwards, 2017). For instance, voice-based AI systems convert speech to text, process it through a language model, and generate synthesised voice responses. This process enables users to interact with AI in a manner that closely resembles human dialogue, thereby expanding its application across education, counselling, and healthcare (Nadarzynski et al., 2024).

The domains of healthcare and caregiving increasingly view AI technologies as tools that enhance operational efficiency and improve the quality of care for patients. M. Kim et al. (2022) suggested that AI-based applications can help caregivers manage tasks and improve work environments. The World Health Organization (WHO, 2024) projected that AI could impact up to 80% of healthcare-related occupations. Sabra et al. (2023) found that nurses generally welcomed AI, though acceptance varied by age, certification, and social status. Bonacaro et al. (2024) argued that integrating AI and big data into nursing care and education could improve diagnostic efficiency and enable personalised learning.

Additionally, Zaboli et al. (2025) reported that AI can aid in emergency decision-making in clinical settings. A systematic review by Borna et al. (2024) found that AI may also alleviate the workload and emotional stress experienced by informal caregivers. Furthermore, AI human devices with GPT-based voice interfaces have shown promise in providing indirect care, such as cognitive assessments and emotional support (Igarashi et al., 2024; Bonacaro et al., 2024).

Despite these advances, real-world applications of AI in care settings remain limited. Ruggiano et al. (2021) critiqued the limited effectiveness of chatbots developed without empirical validation or user-centred design. Recent co-design research has highlighted that technology acceptance in care settings extends beyond functionality to include user-centered design that addresses stakeholders’ real-world needs (Timon et al., 2024). While most studies focus on general attitudes or functional outcomes, few have explored how professional caregivers experience and interpret AI human devices. This gap underscores the need to move beyond quantitative metrics of technology adoption and delve into caregivers’ subjective perspectives in depth.

This study addressed that gap through Q methodology to identify and analyse caregivers’ subjective perceptions of AI–human devices. The study builds on an integrated framework that combines four theoretical perspectives: the Technology Acceptance Model (TAM), the Unified Theory of Acceptance and Use of Technology (UTAUT), Science and Technology Studies (STS), and Human–Machine Communication (HMC) theory.

TAM helps predict user acceptance by emphasising perceived usefulness and ease of use (Davis, 1986), and researchers have broadly applied it in healthcare (Holden & Karsh, 2010). For instance, Haque and Rubya (2023) analysed user reviews of mental health chatbots and found that users appreciated the human-like interactions but often felt frustrated by response errors and perceived misinterpretations of their personalities.

UTAUT extends TAM by incorporating four key constructs: performance expectancy, effort expectancy, social influence, and facilitating conditions (Venkatesh et al., 2003). Yu and Chen (2024) found that among adults over 60, facilitating conditions had the greatest impact on actual use, particularly for those with less technological experience, who relied more on social and effort-related cues.

STS frames technology as a socially constructed entity, shaped by user interpretation (Pinch & Bijker, 1984). It emphasises that technology stems from social experience rather than purely cognitive factors. Ruggiano et al. (2021) noted that chatbot designs that ignore user experience often fall short in effectiveness.

Finally, HMC theory conceptualises AI as an active communicative agent rather than a passive tool. It explores how users form trust, emotional connections, and social meaning in interactions with AI (Guzman & Lewis, 2020). Edwards and Edwards (2017) defined HMC as communication with digital conversational partners, while Lombard and Xu (2021) found that users often anthropomorphise AI agents. Croes and Antheunis (2021) noted that although users initially form social connections with AI, building long-term relationships remains limited.

Through Q methodology, this study categorised caregivers’ subjective perceptions of AI human devices, offering in-depth insights into the emotional and sociotechnical aspects of technology adoption in caregiving. The findings can guide the design of GPT-based voice systems and support the development of emotionally responsive, user-centred AI care technologies.

## 2. Study Method

To explore caregivers’ subjective perceptions of AI human devices in depth, this study adopted Q methodology, a structured approach for capturing subjectivity, grounded in the belief that individuals construct meaning in unique and interpretable ways (Hylton et al., 2018). At its core, Q sorting allows participants to arrange a set of statements according to their self-referential viewpoints, thereby revealing their interpretive frameworks (Brown, 1993). This process respects individual subjectivity and provides a systematic structure for identifying shared patterns of thought among participants (Ramlo, 2015; Van Exel & de Graaf, 2005).

Q methodology effectively classifies subjective viewpoints, making it well-suited to capture caregivers’ experiential perceptions of AI human voice-supported systems for the care of older adults. By focusing on how caregivers interpret and engage with AI technologies based on their lived experiences, the research seeks to uncover individual meaning-making and collective perception structures.

### 2.1. AI Virtual Human

In this study, we examined the AI Virtual Human (AVH) system, which provides distinct functionalities tailored to older adult users and professional caregivers.

Developers designed the AVH for older adults to include features such as companion dialogue (e.g., casual conversation), interactive support tools (e.g., dementia prevention quizzes, read-aloud books, memory aids, sleep and walking reminders), information on chronic disease management, simple dietary recommendations, and guided cognitive and physical activity videos. These features aim to support daily functioning, prevent cognitive decline, and promote independent living among older adults.

Conversely, the caregiver-oriented version of the AVH, accessible via mobile phones and tablets, addresses the practical needs of professional caregivers. Developers structured it to include specialised content distinct from that offered to senior users, with core functions such as AI-guided conversational support, chronic disease management guidelines, dietary planning, and multimedia tools for cognitive and physical exercises. These features enabled caregivers to make on-site decisions and access task-relevant information.

Notably, the caregiver version excluded personal health monitoring and wellness tracking for caregivers and did not integrate with the senior’s health data. Instead, it functioned as a practical tool for managing patient care, reflecting a purpose-built, streamlined design optimised for caregiving tasks.

**Figure 1:**
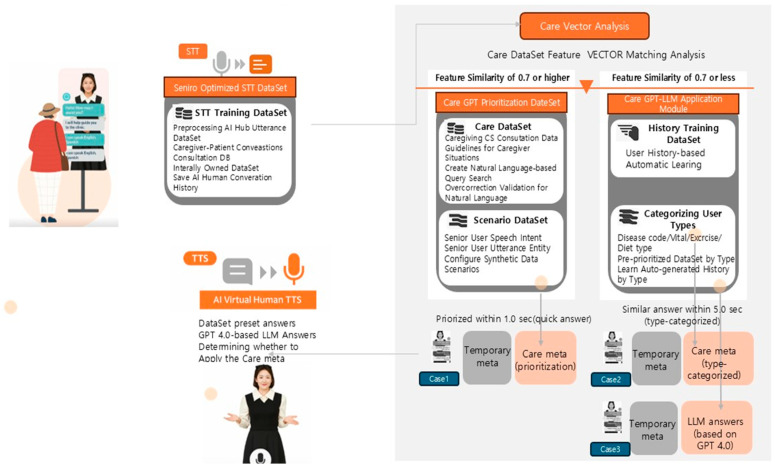
Structural design of the AI Virtual Human (AVH) system.

Furthermore, some caregivers described the device as an informational resource and a form of psychological relief. They viewed it as a ‘socioemotional buffer’ that helped them express emotions and cope with emotional fatigue. This finding suggests that the AVH may contribute to more than just task efficiency and encompass the emotional well-being of caregivers. Figure 1 illustrates the system’s structure and service components analysed in this study.

### 2.2. Research Procedure

**Figure 2:**
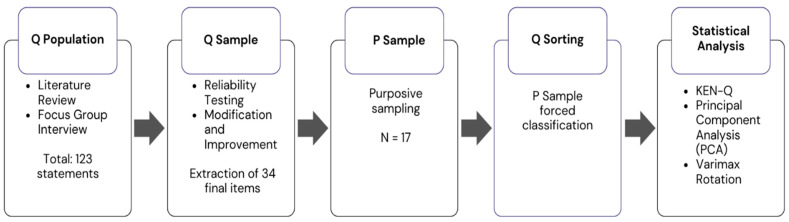
Q methodology steps.

The research procedure consisted of five stages: (1) constructing the Q concourse, (2) selecting the Q sample, (3) selecting the P sample (participants), (4) conducting the Q-sorting process, and (5) analysing the data. Figure 2 provides an overview of the procedure.

#### 2.2.1. Organisation of Q Population (Q Concourse)

The concourse represents the body of opinions, subjectivities, and subjective consciousness commonly shared within a particular culture or society. It encompassed the full range of statements participants could use to express their views on a given topic (H. Kim, 1992). We generated the concourse from literature and a focus group interview (FGI).

First, we reviewed the literature to generate initial statements for the Q concourse. This review focused on domestic and international journal articles as well as master’s and doctoral theses that addressed AI human devices in older adult care. This process yielded 42 preliminary statements.

Next, we used purposive sampling to recruit six nurses, caregivers, and social workers from long-term care hospitals who had prior experience with AI human devices. Participants first completed a pre-interview questionnaire assessing demographic characteristics (age, gender, position), career choice motivation, challenges in caregiving, and key patient relations, followed by the FGI. The FGI participants included three nurses, two caregivers, and one social worker.

We provided each participant with access to the AVH, including detailed usage instructions. We asked participants to use the AVH in their actual caregiving environments for at least 15 days to gain firsthand experience.

We obtained informed consent before the FGI and explained the study’s objectives and procedures. Semi-structured interview questions included: “What expectations did you have before interacting with the AVH, and how did your experience differ,” “How would you describe your overall experience using the program,” “How did the program affect your caregiving work,” “What functions do you think should be added or improved to better support caregivers and older adults,” and “What roles do you think AI-based caregiving programs can play in the future of caring for older adults?”

We continued collecting statements until reaching saturation, finalising 123 statements: 42 derived from the literature review and 81 from the FGI. Table 1 presents the characteristics of the FGI participants.

#### 2.2.2. Selection of the Q Sample

The Q sample refers to a representative subset of statements selected from the broader concourse, designed to reflect the full range of opinions related to the research topic (Brown, 1980). According to Watts and Stenner (2005), a well-constructed Q sample should capture a balanced representation of the discursive landscape, allowing participants’ interpretations to emerge naturally.

We reviewed the 123 statements from the literature review and FGI and categorised them into nine thematic domains: social aspects, practical aspects, healthcare service perspectives, device-related limitations, technological reliability, emotional dimensions, user satisfaction, ethical and value-related concerns, and perceived needs for specific functions. We also classified each statement by tone—positive, negative, or neutral.

We revised or removed statements that were redundant, unclear, or ambiguous. For example, to improve clarity and specificity, we refined the initial statement ‘It would be good if there were functions that protect patient privacy while also providing convenience’, to ‘The AVH should provide personalised care through individual patient data’. Additionally, we removed unclear statements such as ‘Waiting for a slow internet connection feels even more frustrating’, due to poor sentence structure and a lack of direct relevance to the research focus. We refined the remaining pool based on clarity, specificity, neutrality, and representativeness. After expert review by two Q methodology specialists, we selected a final set of 34 statements for the Q sample.

#### 2.2.3. Composition of the P Sample

The P sample consists of participants who sort the Q-sample statements based on their subjective viewpoints (H. Kim, 2007). Since Q methodology does not aim for demographic generalisability, it does not require statistical representative sampling (Hylton et al., 2018). Even a single participant can suffice when the goal is to explore internal cognitive structures (Brown, 1980). Rather than measuring differences between individuals, Q methodology focuses on how individuals interpret and assign meaning to an issue. Therefore, participants must closely and meaningfully engage with the research topic (H. Kim, 2007).

For this study, we used purposive sampling to select 17 participants, including caregivers, nurses, and social workers, who had at least 15 days of experience using the AVH in real care settings. We prioritised participants with direct relevance to the phenomenon under investigation.

The P sample included individuals with diverse demographic characteristics such as age, gender, occupation, career motivations, caregiving challenges, and the attributes of their care recipients. We considered these background variables as key factors influencing subjective perspectives. Table 2 shows the characteristics of participants categorised according to the three factors identified through subsequent Q-factor analysis.

We also included five of the six FGI participants in the P sample (P1, P2, P5, P8, P16). In Q methodology, including the same participants in the initial in-depth interview and Q-sorting is a methodologically sound approach. This approach enables a deeper understanding of participants’ subjective experiences and perceptions, thereby increasing data consistency and depth (de Guzman et al., 2012). Additionally, having the same participants evaluate the statements derived from the initial interview in Q-sorting ensures data continuity.

The Ethics Committee of the authors’ Institutional Review Board approved this study on 28 August 2024 (approval No. DUIRB2024-08-02).

#### 2.2.4. Q Sorting

Q sorting is the process by which participants rank the Q sample statements based on their subjective judgments. This procedure reveals each individual’s internal perception structure and values (Brown, 1980).

**Figure 3:**
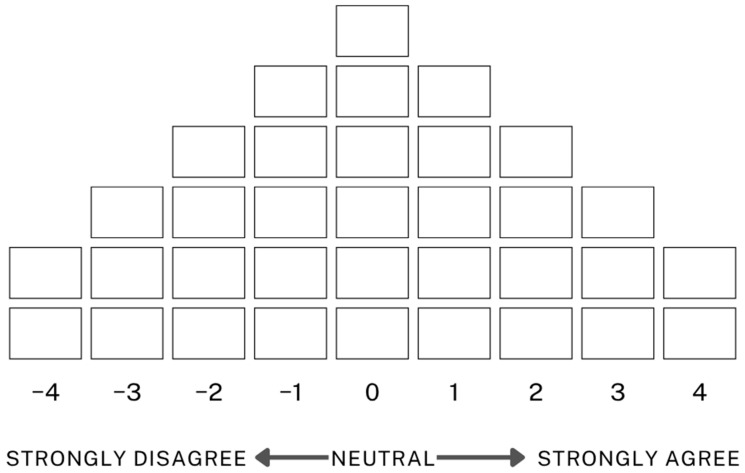
Q-sort distribution grid.

Participants conducted Q-sorting in person, with researchers directly explaining the procedure and method to each participant and remaining present throughout the entire process. Participants completed the Q-sort individually using a standardised distribution grid (see Figure 3) provided by the researchers. The grid followed a forced-choice distribution, a quasi-normal distribution ranging from −4 to +4, where participants sorted statements based on their degree of agreement or disagreement with each statement. This method allowed us to uncover each participant’s unique structure of subjectivity (Brown, 1993). The forced distribution also ensured that participants could distinguish between statements meaningfully based on their relative importance.

Following the completion of Q-sorting, participants wrote why they chose their highest-ranked (+4) and lowest-ranked (−4) statements. Researchers then conducted individual post-sorting reviews with each participant, examining their written explanations and asking clarifying questions where necessary. These qualitative data served as key evidence for interpreting each factor type.

#### 2.2.5. Data Analysis

We analysed data using Ken-Q analysis software (version 1.3.1), which supports the interpretive construction of subjectivity types based on similarities and differences in Q-sort patterns. Although classical Q methodology traditionally employs centroid extraction and manual rotation (Watts & Stenner, 2005), this study adopted principal component analysis (PCA) with varimax rotation as implemented in the Ken-Q software.

PCA offers computational stability, and researchers widely use it in contemporary Q studies, producing results comparable to those derived from centroid extraction (Brown, 1993; Van Exel & de Graaf, 2005; Zabala, 2014; Ramlo, 2015). The combination of PCA and varimax rotation clearly distinguished participants’ perceptual patterns in the data, providing a statistically coherent structure that facilitated the theoretical interpretation and classification of caregivers’ subjective viewpoints. Recent Q methodology studies across health, psychology, and the social sciences have widely used this combination due to its reproducibility and interpretive clarity (Churruca et al., 2021).

We retained factors in accordance with standard practices in Q methodology. Specifically, we selected factors that (1) had eigenvalues greater than 1.0, (2) included at least two significantly loading Q-sorts (*p* < 0.01), and (3) demonstrated theoretical interpretability. Among factors exceeding the eigenvalue threshold, we prioritised those with higher eigenvalues and clearer loading structures, provided they met the interpretability criterion. These criteria are consistent with established methodological guidelines (Brown, 1978; Van Exel & de Graaf, 2005; Akhtar-Danesh, 2017; Damio, 2018). We treated factor loadings of ±0.43 or higher as statistically significant (*p* < 0.01) and calculated this threshold using the formula 2.58 × (1/√N), where *N* is the number of Q statements (N = 34 in this study) (Brown, 1980; Van Exel & de Graaf, 2005). In Q methodology, factor loadings indicate the degree to which each Q-sort contributes to or represents a given factor, rather than merely reflecting statistical correlations in the conventional sense (Brown, 1993).

We interpreted each factor by examining standardised z-scores and Q-sort values to identify distinguishing statements that significantly differentiated one factor from the others, as well as characterising statements with the highest or lowest z-scores within each factor that represented participants’ shared core viewpoints (Brown, 1980; Watts & Stenner, 2005). These statistical measures enabled us to identify consensus and divergent perspectives across the different types. We also incorporated participants’ qualitative explanations of the statements that they highest-ranked (statements with which they most agreed) and lowest-ranked (statements with which they most disagreed). These narratives served as key evidence for interpreting the subjective orientation of each type (Van Exel & de Graaf, 2005).

In Q methodology, a ‘factor’ represents a group of participants who sorted the statements in similar ways. Each factor indicates a distinct subjective viewpoint or perception type. In this study, factor analysis yielded three factors (Type 1, Type 2, and Type 3), with each factor representing a distinct perception type regarding the AVH.

## 3. Study Results

### 3.1. Q-Factor Analysis Results

Table 3 shows that all extracted factors had eigenvalues greater than 1.0. Specifically, Factor 1 recorded an eigenvalue of 4.01352, Factor 2 registered 2.122581, and Factor 3 yielded 1.904245. These three factors collectively explained approximately 47% of the cumulative variance.

Factor analysis revealed three distinct perception types. A higher factor weight indicated a stronger representativeness of the participant within each type. Q-sorts with the highest factor weights defined Type 1, explaining 24% of the total variance. Type 2 and Type 3 each explained 12% and 11% of the variance, respectively. Together, the three types accounted for 47% of the total variance, indicating an adequate and interpretable factor solution (Brown, 1980; Van Exel & de Graaf, 2005).

Table 4 displays the correlation coefficients among the factors. The correlation between Factor 1 and Factor 2 was −0.0831, between Factor 1 and Factor 3 was 0.0551, and between Factor 2 and Factor 3 was 0.0564.

Table 5 presents the z-scores and Q-sort values for each type. An asterisk (*) marks statements that serve as distinguishing statements, indicating statistically significant difference at the *p* < 0.01 level.

### 3.2. Perception Type Characteristics

#### 3.2.1. Type 1: Active Acceptors

Type 1, the ‘Active Acceptors’, demonstrated a high level of trust in AVH devices and a strong willingness to integrate them actively into patient care. Participants in this group adopted a proactive approach to incorporating AI technologies into their caregiving practices.

They most strongly agreed with the statements: ‘Sensor integration for real-time patient monitoring would be useful’ (Q25, Z = 1.98) and ‘Entertainment features help reduce stress when caring for patients’ (Q12*, Z = 1.74). In contrast, they most strongly disagreed with ‘I cannot trust the AVH system’ (Q8, Z = −1.57) and ‘The mechanical nature of the device makes it feel unfamiliar and impersonal’ (Q27*, Z = −1.85).

Beyond practical functions, Type 1 caregivers also distinctively valued the emotional dimension of AI interaction. They agreed that ‘Interaction with the AVH lifts my mood’ (Q9*, Z = 1.11), appreciated the ability to confide in the device about matters they found difficult to discuss with others: ‘I can say things to the AVH that I can’t easily say to others’ (Q16*, Z = 1.44), and found comfort in the empathetic responses from the AVH when sharing caregiving burdens (Q13*, Z = 1.41).

P11, classified under this type, shared the following experience: ‘When using the AI device, we often played trot music with the older adults. It really helped relieve stress for us. I was surprised when the patients clapped and even sang along’. P11 also remarked, ‘Care becomes more effective when the patient’s condition is easy to monitor. It would be great if the device had a sensor-based real-time monitoring feature. That way, I could respond promptly to changes in the patient’s condition’.

These narratives reflect the open and flexible attitudes of Type 1 caregivers towards AVH devices. They view such technologies as tools and collaborative partners that can support their caregiving responsibilities. Their responses reveal a practical commitment to enhancing care quality through AI. Based on these features, we labeled this type ‘Active Acceptors’.

#### 3.2.2. Type 2: Improvement Seekers

The key characteristic of the ‘Improvement Seekers’ (Type 2) is a low level of trust in AVH devices and the belief that these systems require significant improvement before they can function effectively in real-world care settings. Participants in this group expressed cautious and critical views about the current capabilities of AVH systems.

This type most strongly agreed with the statements, ‘It takes too long for the AVH to recognise speech, which is inconvenient’ (Q15, Z = 1.83) and ‘I wouldn’t use the system without financial support’ (Q10*, Z = 1.69). Conversely, they most strongly disagreed with ‘The AVH helps me respond effectively to changes in the patient’s condition’ (Q6, Z = −1.89) and ‘Interacting with the AVH feels like receiving counselling’ (Q3*, Z = −2.03).

Participant P7, categorised under this type, commented the following:

It’s frustrating how long it takes for the device to recognise speech, and overall, I don’t think it helps much in daily caregiving. It takes time to activate and process commands properly, and the device lacks sufficient connectivity with medical staff to be useful in responding to changes in a patient’s condition.

Similarly, P1 commented, ‘If the device integrated a patient monitoring sensor, it would be easier to manage medication times or check meals on schedule. But as it stands, the system is limited and would need significantly more data accumulation to be useful.’

Unlike Type 1 participants, Type 2 participants distinctly rejected the emotional and practical values of AI interaction. They strongly disagreed that they could confide in the device about personal matters: ‘I can say things to the AVH that I can’t easily say to others’ (Q16*, Z = −1.66). They also found it inconvenient for accessing real-time caregiving knowledge: ‘It’s convenient to access knowledge needed on the spot while caregiving’ (Q22*, Z = −0.79). Despite their critical stance, they did not attribute their concerns to the mechanical nature of the device (Q27*, Z = −1.02).

These responses reflect a critical and discerning stance towards AVH devices. Caregivers in this group questioned the practical utility of the technology in its current form and called for substantial improvements in functionality, responsiveness, and real-time adaptability. They prioritised operational effectiveness and situational fit over adopting new technologies for their novelty. Based on these characteristics, we labeled this type the ‘Improvement Seekers’.

#### 3.2.3. Type 3: Emotional Support Seekers

The defining feature of Type 3, the ‘Emotional Support Seekers’, is their emotional satisfaction when using AI devices as a form of relief and self-expression. Participants in this group perceived the AVH as a caregiving tool and source of personal comfort.

The statements with which this type most strongly agreed were: ‘I can say things to the AVH that I can’t easily say to others’ (Q16*, Z = 2.28) and ‘Interacting with the AVH feels like receiving counselling’ (Q3*, Z = 1.57). In contrast, they most strongly disagreed with ‘I cannot trust the AVH’ (Q8, Z = −1.83) and ‘The AVH helps me respond effectively to changes in the patient’s condition’ (Q6, Z = −2.00).

P3, in this group, stated

The AI responses were often empathetic and closely matched how I felt, which made me feel understood and emotionally uplifted. I was able to share personal or deeply held thoughts, which brought a sense of relief. While talking to the AI helped shift my mood during moments of sadness, I also felt the need to approach it carefully so as not to become emotionally dependent on it. Still, it helped me recover from temporary emotional lows.

Similarly, P9 shared

When I talked to the AI about difficult experiences, it responded empathetically and answered my questions in detail, which built trust. But at the same time, I still felt it was ‘just a machine’, so the emotional connection was limited and didn’t fully relieve my stress.

Type 3 participants distinctively prioritised emotional connection over functional features. Unlike Type 1, they showed neutral attitudes towards practical enhancements such as ‘Sensor integration for real-time patient monitoring would be useful’ (Q25*, Z = −0.04). They also acknowledged the mechanical limitations of AI, agreeing with the statement, ‘The mechanical nature of the device makes it feel unfamiliar and impersonal’ (Q27*, Z = 0.86), and even preferred direct internet searches for efficiency: ‘Searching the internet directly is faster and easier’ (Q26*, Z = 1.01). Despite recognising these practical and technological constraints, they continued to value the emotional support the device provided.

Caregivers in this group sought emotional support from the AVH to manage the psychological demands of caregiving. They perceived the device as a potential source of emotional companionship and a tool for self-regulation. Their responses suggest that AI systems could foster psychological resilience. Based on these characteristics, we labeled this group the ‘Emotional Support Seekers’.

## 4. Discussion

This study explored how caregivers for older adult patients with chronic illnesses subjectively perceived the AI Virtual Human (AVH) application. The analysis identified three distinct perception types: Active Acceptors (Type 1), Improvement Seekers (Type 2), and Emotional Support Seekers (Type 3).

Participants in the Active Acceptors group demonstrated high trust in AVH caregiving devices and a strong intention to use the technology proactively for patient-centred care. Their acceptance aligns with the Technology Acceptance Model (TAM), particularly the constructs of perceived usefulness and perceived ease of use. Consistent with Holden and Karsh’s (2010) argument that these two variables significantly influence acceptance of technology in clinical settings, participants in this type expressed strong motivation to adopt AVH devices. They valued the technology primarily for its utility in monitoring patient conditions and relieving caregiving stress. Notably, they did not define ‘usefulness’ in terms of personal convenience but through the lens of their caregiving responsibilities. Many expressed this view through statements such as ‘It’s good that I can use the device for my patients’, while rarely referencing personal benefits. This pattern suggests that they framed usefulness in terms of enhancing caregiving performance and patient outcomes.

Active Acceptors appear to view the AVH device through the lens of technological determinism. They believe that advancements in technology inherently improve the quality of caregiving work. Their proactive and optimistic stance reflects confidence in technology’s transformative potential. Ultimately, Active Acceptors combine high trust in AVH devices and patient-centred values, driven by the core constructs of TAM and a belief in technology’s ability to elevate care.

This study extends TAM by reframing perceived usefulness as a socially embedded construct grounded in caregiving ethics rather than individual convenience. In doing so, it highlights how caregivers interpret usefulness through relational and moral dimensions, expanding TAM beyond cognitive and efficiency-based parameters to include ethical and affective aspects of technology adoption in healthcare.

Type 2, the Improvement Seekers, expressed low trust in the AVH device while maintaining a primary focus on patient care. They criticised the device’s current performance and usability, which resonates with the Unified Theory of Acceptance and Use of Technology (UTAUT) (Venkatesh et al., 2003). Specifically, these participants reported low performance expectancy and high effort expectancy, two UTAUT constructs that reduce behavioural intention to adopt new technologies. This finding aligns with Kwak et al. (2022), who showed that both constructs predict AI healthcare technology use among nursing students.

Improvement Seekers emphasised that the meaningful integration of AI into caregiving requires greater precision, real-time responsiveness, and seamless system integration. Their views rejected technological determinism and instead highlighted social constructivist thinking, where users define the value and relevance of technology based on context and social negotiation (Pinch & Bijker, 1984). These caregivers demand smarter, more context-sensitive features. For them, adoption does not hinge on novelty but on how well the device fits within the actual caregiving environment and the user’s experience.

Beyond reaffirming the UTAUT framework, this study extended it by embedding performance and effort expectancy in a social constructionist perspective. Improvement Seekers illustrated how these constructs operate as individual predictors and socially negotiated evaluations that depend on context-specific constraints, institutional norms, and collective feedback loops between caregivers and technology developers. This integration bridges UTAUT and STS, positioning technology acceptance as a functional and socio-contextual process.

The final type of caregiver in our study, the Emotional Support Seekers (Type 3), exhibited neutral or low trust in the AVH and used it primarily to regulate their emotions rather than deliver patient care. They found comfort in confiding in the device, using it as a tool for emotional expression and relief. This perspective extends the traditional TAM framework by introducing emotional usefulness as a parallel to perceived usefulness. For these caregivers, the AVH served as an emotional support system, helping to alleviate stress and facilitate interpersonal communication.

Participants’ narratives indicated that the AVH provided comfort during emotionally difficult moments, helping them recover from feelings of isolation and burnout. These findings align with recent studies (Haque & Rubya, 2023; Siddals et al., 2024; Merx et al., 2025), which found that emotional comfort and psychological safety significantly influenced user attitudes towards AI technologies. We can interpret this pattern using the Social Construction of Technology (SCOT) framework (Bijker, 1997), which emphasises how users reshape technology’s meaning based on their needs and social context. In this case, Emotional Support Seekers reinterpret the AVH as a socioemotional support system, diverging from its original function as a caregiving tool.

This reconceptualisation also aligns with Human–Machine Communication (HMC) theory, which views AI as a relational and communicative partner (Guzman & Lewis, 2020). For instance, Li et al. (2024) found that patients’ acceptance of AI-driven medical advice depended heavily on the system’s communication style and perceived social responsiveness. Emotional Support Seekers demonstrate this point by accepting and continuing to use the AVH because it offered emotional connection, comfort, and psychological relief.

This study expands HMC by situating emotional interaction within the caregiving relationship, where AI acts as a relational mediator that fosters empathy, containment, and self-regulation. Unlike prior HMC studies that focused on general communication or companionship, our findings indicate that emotional trust and therapeutic presence can also emerge in professional caregiving contexts, suggesting a new dimension of relational resilience in human–AI interactions.

In summary, Emotional Support Seekers perceived the AVH as a functional tool for enhancing patient care and a companion that supported their emotional well-being. Their experiences illustrate the evolving social role of AVH in caregiving, shifting from a task-oriented instrument to an emotionally meaningful partner. These findings suggest that the successful implementation of AVH caregiving technologies in real-world settings requires sensitivity to the distinct needs and orientations of each caregiver type.

For Active Acceptors, maintaining engagement relies on strengthening system reliability and interoperability, alongside the continuous incorporation of on-site feedback to improve usability and trust. For Improvement Seekers, technological sophistication and user-centred validation processes are crucial, with feedback loops between caregivers and developers enhancing practical applicability and contextual fit. Finally, Emotional Support Seekers require empathy-based conversational algorithms and emotional support modules to mitigate compassion fatigue and psychological stress. Collectively, these implications underscore the need to design AVHs that reflect the emotional, cognitive, and contextual dimensions of caregiving practice.

## 5. Limitations and Future Research Directions

While this study offers valuable insights into caregivers’ subjective perceptions of AVHs, it also has several limitations that provide direction for future research.

First, the sample consisted primarily of caregivers working in long-term care hospitals and facilities located in one geographic area. Therefore, regional characteristics may have influenced their perceptions. Future research should conduct cross-regional comparative studies or incorporate more representative quantitative approaches to enhance generalisability. While this study focused on caregivers’ subjective perceptions based on their actual experiences using AI caregiving devices, future research should include participants who are less familiar with AI technologies to explore how initial exposure, unfamiliarity, and learning processes shape perceptions and acceptance of AI in caregiving contexts.

Second, the average usage period for the AVH was approximately 15 days, and researchers did not impose a standardised usage protocol. Some participants used the device jointly with patients, while others used it independently. These varied contexts may have affected how participants experienced the system. Future studies should consider controlling for usage environments or providing clear usage guidelines to facilitate more accurate comparisons across perception types.

Third, the sample included more female than male caregivers. This gender imbalance reflects broader structural trends in the caregiving profession, but it limited the study’s ability to examine gender-based differences in technology acceptance. Future research should prioritise gender-diverse sampling to address this gap.

Despite these limitations, this study makes important contributions. It revealed that AI caregiving technologies support task-based functions and caregivers’ emotional well-being and psychological resilience. Researchers should expand future work to include more caregiving professions and regions, while also developing new models of technology acceptance that incorporate emotional and cultural dimensions. A mixed-methods approach that integrates Q methodology with quantitative assessments may offer a more comprehensive understanding of how users perceive and engage with AI systems.

## 6. Conclusions

This study employed Q methodology to explore caregivers’ subjective perceptions of AI Virtual Human (AVH) devices in the care of older adults and identified three distinct perception types: Active Acceptors, Improvement Seekers, and Emotional Support Seekers. The results indicate that caregivers’ acceptance of AI extends beyond functional and instrumental efficiency to include emotional connection, relational meaning, and contextual interpretation, reflecting the complex and human-centred nature of technology use in caregiving.

By integrating theoretical perspectives from the Technology Acceptance Model (TAM), the Unified Theory of Acceptance and Use of Technology (UTAUT), Science and Technology Studies (STS), and Human–Machine Communication (HMC), this study contributes to a more comprehensive understanding of human–AI interactions in care contexts.

Practically, the findings emphasise the need for user-centred AI design and policy development that account for caregivers’ emotional and experiential realities. Future studies should include participants with limited or no prior experience with AI to capture a broader range of perceptions and investigate cross-cultural and longitudinal variations in AI acceptance within caregiving settings.

## Figures and Tables

**Table 1:** Demographic characteristics of participants in the focus group interview.

Participant Label (Number)	Age (Gender)	Position	Career Choice Motivation	Challenges	Average Patient Age	Key Patient Relations
A (P16)	48 (F)	Nurse	Job stability	Physical strain/Emotional difficulty	80~89	Trust/Emotional support/Communication
B (P2)	68 (F)	Caregiver	Desire to help others	Emotional difficulty	60~69	Communication
C (P1)	56 (F)	Nurse	Desire to help others	Emotional difficulty	80~89	Trust
D (P5)	55 (M)	Social Worker	Social recognition/Acquisition of professional skills	Relationship with patients’ families	70~79	Trust
E (P8)	51 (F)	Caregiver	Desire to help others	Emotional difficulty	80~89	Emotional support
F (-)	40 (F)	Nurse	Desire to help others/Job stability	Emotional difficulty	80~89	Communication

**Table 2:** Demographic characteristics of the P sample.

Type	P Sample No.	Factor Weight	Age (Gender)	Position	Career Choice Motivation	Challenges	Average Patient Age	Key Patient Relations
Type 1 (N = 9)	P11	0.7901	46 (F)	Caregiver	Meaningful experience	Physical strain	60~89	Trust
	P17	0.7695	50 (F)	Nursing Assistant	Job stability	Communication issues with patients	70~79	Consistent care
	P15	0.7141	45 (F)	Nurse	Acquisition of professional skills	Communication issues with patients	60~89	Emotional support
	P16	0.6999	48 (F)	Nurse	Job stability	Physical strain/Emotional difficulty	80~89	Trust/Emotional support/Communication
	P2	0.6934	68 (F)	Caregiver	Desire to help others	Emotional difficulty	60~69	Communication
	P13	0.5557	42 (F)	Caregiver	Meaningful experience	Communication issues with patients	80~89	Trust
	P6	0.5037	43 (F)	Nurse	Job stability/Desire to help others	Emotional difficulty	80~89	Communication
	P4	0.3834	61 (F)	Caregiver	Desire to help others	Communication issues with patients	80~89	Trust
	P10	−0.3381	51 (F)	Caregiver	Desire to help others	Physical strain	80~89	Trust
Type 2 (N = 4)	P7	0.7963	43 (F)	Caregiver	Job stability	Time management	70~79	Communication
	P1	0.7	56 (F)	Nurse	Desire to help others	Emotional difficulty	80~89	Trust
	P8	−0.635	51 (F)	Caregiver	Desire to help others	Emotional difficulty	80~89	Emotional support
	P5	0.5693	55 (M)	Social Worker	Social recognition/Acquisition of professional skills	Relationship with patients’ families	70~79	Trust
Type 3 (N = 4)	P3	0.7286	52 (F)	Caregiver	Job stability	Physical strain/Communication issues with patients	70~79	Trust/Communication
	P14	0.6337	58 (F)	Caregiver	Meaningful experience/Job stability	Communication issues with patients	80~89	Trust
	P9	0.5368	50 (F)	Nursing Assistant	Job stability	Emotional difficulty	70~79	Trust
	P12	0.3333	63 (F)	Caregiver	Meaningful experience/Social recognition	Physical strain	80~89	Trust

**Table 3:** Eigenvalues and explanatory variances in the sorting of three factors.

Content	1	2	3
Eigenvalues	4.01352	2.122581	1.904245
Explained Variance (%)	24	12	11
Cumulative Explained Variance (%)	24	36	47

**Table 4:** Correlation coefficients between factors.

Type	1	2	3
1	1	−0.0831	0.0551
2		1	0.0564
3			1

**Table 5:** Z-scores and Q-sort values of Q statements by factor type.

No.	Statement	1		2		3	
		Z-Score	Q-Sort Value	Z-Score	Q-Sort Value	Z-Score	Q-Sort Value
1	The AVH feels too heavy to use comfortably.	−1.18	−3	−0.11	0 *	−1.48	−3
2	The character in the AVH feels like I’m speaking to a real person.	−0.96	−2	−1.21	−3	−0.11	0 *
3	Interacting with the AVH feels like receiving counselling.	0.01	0 *	−2.03	−4 *	1.57	4 *
4	The AVH is simple to operate.	0.3	1 *	−0.37	−1	−0.82	−2
5	I don’t know who to turn to when problems occur while using the AVH.	−0.18	−1	−0.33	−1	0.6	1 *
6	The AVH helps me respond effectively to changes in the patient’s condition.	0.28	1 *	−1.89	−4	−2	−4
7	It is difficult to use the AVH for medical purposes.	0.1	0 *	0.69	1	−0.84	−2 *
8	I cannot trust the AVH system.	−1.57	−4	−0.28	0 *	−1.83	−4
9	Interaction with the AVH lifts my mood.	1.11	2 *	−1	−2	−0.87	−3
10	I wouldn’t use the system without financial support.	−0.97	−2	1.69	4 *	−0.79	−2
11	It’s hard to get the answers I want from the AVH.	−0.21	−1	0.49	1	0.26	1
12	Entertainment features help reduce stress when caring for patients.	1.74	4 *	−0.52	−1	−0.46	−1
13	Sharing my struggles with the AVH helps relieve stress because it shows empathy.	1.41	3 *	−1.04	−3	−1.59	−3
14	Functional issues make the AVH difficult to use.	−1.23	−3 *	0.24	1	−0.1	0
15	It takes too long for the AVH to recognise speech, which is inconvenient.	−1.05	−2 *	1.83	4	1.02	2
16	I can say things to the AVH that I can’t easily say to others.	1.44	3 *	−1.66	−3 *	2.28	4 *
17	The AVH asks too many questions, making it boring.	−1.25	−3 *	−0.34	−1	−0.29	−1
18	It feels like the system lacks sufficient big data.	−0.93	−1 *	0.77	1	0.29	1
19	The AVH should offer features for emergencies.	0.43	2	0.94	2	1.13	3
20	Voice recognition often fails with accents or dialects, which is frustrating.	−1.15	−2 *	0.56	1	1.14	3
21	The device is helpful in suggesting responses to sudden changes in a patient’s condition.	−0.01	0	−0.14	0	0.18	0
22	It’s convenient to access the knowledge needed on the spot while caregiving.	1.49	3	−0.79	−2 *	1.02	2
23	Existing AI-based real-time platforms are more effective than the AVH.	−0.07	0	0.12	0	−0.25	0
24	It would be helpful if the system could store and manage patient information.	0.81	2	0.92	2	−0.51	−1 *
25	Sensor integration for real-time patient monitoring would be useful.	1.98	4	1.63	3	−0.04	0 *
26	Searching the internet directly is faster and easier.	−0.6	−1	−0.11	0	1.01	2 *
27	The mechanical nature of the device makes it feel unfamiliar and impersonal.	−1.85	−4 *	−1.02	−2 *	0.86	2 *
28	The AVH should provide personalised care through individual patient data.	1.04	2	0.98	3	0.61	1
29	Caregivers can collaborate with the AVH to improve work efficiency.	0.3	1 *	−0.75	−2	−0.28	−1
30	The AVH should be able to connect directly with medical staff in emergencies.	0.24	0	0.23	0	0.55	1
31	It would be better if the AVH could assist with medication management.	0.33	1 *	0.92	2	−0.76	−2
32	The AVH should track eating habits and offer appropriate advice.	0.32	1	0.86	2	0.21	0 *
33	The AVH must strictly protect patient anonymity.	0.19	0 *	1.01	3	1.04	3
34	The AVH’s design should be senior-friendly.	−0.28	−1	−0.3	−1	−0.73	−1

Note. * statistically significant difference (*p* < 0.01) defining a given factor type.

## Data Availability

The data are available from the corresponding authors upon reasonable request.
